# PM_2.5_ in Urban and Rural Nursery Schools in Upper Silesia, Poland: Trace Elements Analysis

**DOI:** 10.3390/ijerph120707990

**Published:** 2015-07-14

**Authors:** Anna Mainka, Elwira Zajusz-Zubek, Konrad Kaczmarek

**Affiliations:** 1 Department of Air Protection, Silesian University of Technology, 22B Konarskiego St., Gliwice 44-100, Poland; E-Mail: Elwira.Zajusz-Zubek@polsl.pl; 2 Institute of Mathematics, Silesian University of Technology, 23 Kaszubska St., Gliwice 44-100, Poland; E-Mail: Konrad.Kaczmarek@polsl.pl

**Keywords:** indoor air quality, children, PM_2.5_, trace elements, enrichment factor (EF), principal component analysis (PCA)

## Abstract

Indoor air quality (IAQ) in nursery schools is an emerging public health challenge. Particular attention should be paid to younger children, because they are more vulnerable to air pollution than older children. Among air pollutants, fine particulate matter (PM_2.5_) is of the greatest interest mainly due to its strong association with acute and chronic effects on children’s health. In this paper, we present concentrations of PM_2.5_ and the composition of its trace elements at naturally ventilated nursery schools located in the area of Gliwice, Poland. The nursery schools were selected to characterize areas with different degrees of urbanization and traffic densities during the winter and spring seasons. The results indicate there is a problem with elevated concentrations of PM_2.5_ inside the examined classrooms. The children’s exposure to trace elements was different based on localization and season. PM_2.5_ concentration and its trace element composition have been studied using correlation coefficients between the different trace elements, the enrichment factor (EF) and principal component analysis (PCA). PCA allowed the identification of the three components: anthropogenic and geogenic sources (37.2%), soil dust contaminated by sewage sludge dumping (18.6%) and vehicular emissions (19.5%).

## 1. Introduction

The fact is that people spend most of their time indoors. Fortunately, over the last decade, an awareness of the higher concentrations of air pollutants in indoor environments compared to outdoors has increased. With the growing interest in studying people’s exposure to air pollutants, attention should focus on children, particularly younger ones. The exposure of preschool children to air pollutants could represent a very interesting study for three main reasons: (a) children are particularly vulnerable to the harmful effects of air pollution because of immature lung defences, narrower airways, higher inhalation rates and a higher metabolic rate of oxygen consumption per unit of body weight [1,2,3]; (b) younger children spend more time in preschool than in any other indoor environment besides the home; (c) other factors, such as furnishing, sorptive materials (carpets, toys and bedcovers) and children’s activities, influence the concentrations of air pollutants, and thus the IAQ (indoor air quality) in preschools is different from that in primary schools [3,4].

Epidemiological studies have consistently shown an association between atmospheric particle pollution and the number of respiratory and cardiovascular diseases [5,6,7]. Much research has pointed towards PM_2.5_, which is able to penetrate deep into the human lungs and which usually contains hazardous substances [8]. Over the last decade, a considerable number of studies regarding PM levels in schools have been presented, e.g., the review by Siv *et al.* [9]; only a few concerned preschools [3,10,11,12,13,14]. However, there is a shortage of data on PM compositions in preschools [15], even though the chemical composition of PM, especially the concentration of potentially toxic trace elements, plays a decisive role in the assessment of air pollution and its hazard to children’s health.

Fine particles can easily enter a child’s body during their playing activities. They are deposited in the lower respiratory tract (tracheobronchial region and alveoli), thereby having a greater effect on causing or aggravating respiratory diseases [16]. Hazardous elements can adsorb onto the surface of these particles, contributing to important adverse health effects. For example, a high intake of heavy metals by children has been associated with mood swings, poor impulse control and aggressive behaviour, along with a poor attention span, depression and apathy, disturbed sleep patterns and impaired memory and intellectual performance [17].

It is known that there are several different sources of trace elements in indoor dust and that they depend on the condition and location of the building, the activities of occupants and the outdoor sources [15]. Industry and city traffic, top soil and building materials, especially during renovation, are among the sources of trace elements in preschools [18]. Trace elements may be transported by means of ventilation systems and brought from outdoors by the children’s movements.

The aim of this study was to determine the concentrations of selected trace elements (As, Cd, Cr, Cu, Fe, Mn, Ni, Pb, Sb, Se and Zn) in the fine particulate matter (PM_2.5_) collected inside and outside four preschools located in the area of Gliwice, Poland. To date, there has been no study comparing urban and rural preschools which considers the PM_2.5_ composition in terms of children’s exposure; this study aims to cover this gap.

## 2. Material and Methods

**Figure 1:**
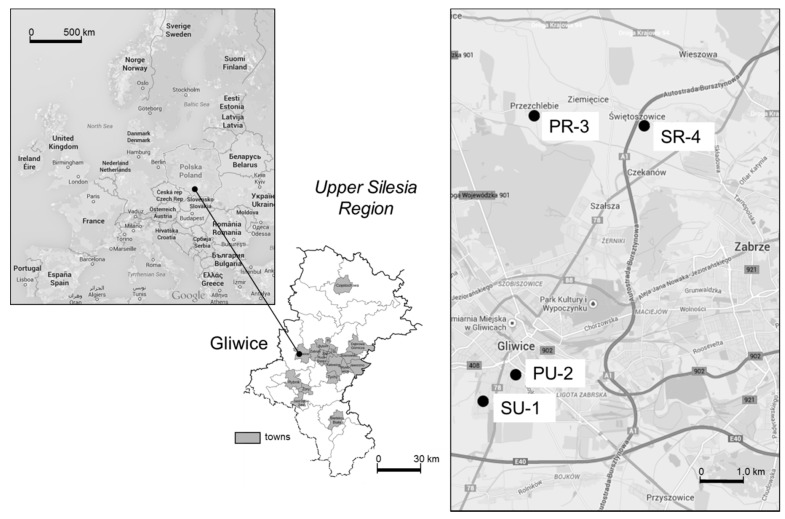
Map showing the sampling sites, *SU-1* residential area; *PU-2* city centre; *PR-3* rural area—village; *SR-4* rural area—highway (screens) (Map data: ©2015 Google, ORION-ME).

This study was carried out between 9 December 2013 and 23 May 2014 at four nursery schools in the area of Gliwice, located in the Upper Silesia region in the southern part of Poland (Figure 1). Broadly, there are 4.5 million residents in the region, and approximately 151,000 children participating in preschool education.

### 2.1. Sampling Sites and Buildings

Two of the nursery schools are located in an urban area and two in a rural area. The first building, labelled SU-1 (Sikornik district, Urban area 1), is located in the residential area to the southwest of the city centre, next to an air quality monitoring station. The second is located in an urban traffic area, and is labelled PU-2 (Pszczynska Street, Urban area 2). The front facade of the PU-2 building is located 50 m from the street, with heavy traffic reaching 2400–2800 vehicles per hour [19]. Between the PU-2 building and the street, there is parking space available which enables the flow of air from the traffic in the street. Two more buildings are located in the rural areas in the northeast approximately 10 km from Gliwice. The location was selected according to the dominant wind direction (207°) in the region. The PR-3 side (Przezchlebie village, Rural area 3) represents typical rural localization without industrial activity or heavy traffic, while the SR-4 building (Świętoszowice village, Rural area 4) is located 50 m from highway A1 (a road section opened since 2011). The building is separated from the highway by highway screens. All the nursery schools are located in detached buildings, all of which underwent the process of thermal efficiency improvement, completed in 2007 (PU-2), 2008 (SU-1) or summer 2013 (PR-3 and SR-4). During the thermal insulation process, the natural ventilation using the air duct systems of the buildings was left unchanged. Consequently, the IAQ is mostly ensured by means of stack ventilation and airing through open and unsealed windows.

The buildings have kitchens which use gas stoves located on the first (SU-1 and SR-4) or ground floors (PU-2 and PR-3). The urban nursery schools had 100 (SU-1) and 150 (PU-2) children divided by age into four or six different classrooms, respectively. Meanwhile, in the rural buildings, 54 (PR-3) and 56 (SR-4) children were divided into three groups. In each nursery school, the measurements were conducted in older (I) and younger (II) children’s classrooms.

### 2.2. Sampling Method

Indoor and outdoor samples of PM_2.5_ were collected and identified following the reference procedure PN-EN 12341:2014 [20]. The samples were collected during each working day from Monday morning to Friday afternoon between 7:30 to 15:30. Paralleled samples of PM_2.5_ were collected from each playground and inside selected classrooms during the occupation period. There were two one-week sessions in each nursery school during winter and spring seasons. Inside the classrooms, the PM_2.5_ samples were collected using a three-stage impactor (Dekati^®^ PM10 impactor, Finland) with a flow-rate of 30 L/min. The cut-off diameters of the impactor stages were 10, _2.5_ and 1 µm. The collection substrates on the impaction stages were polycarbonate membranes (Nuclepore Whatman, diameter 25 mm). The backup filter material was made of Teflon (Teflo Pall, diameter 47 mm). The collection of outdoor samples was performed using quartz microfibre filters (QM-A Whatman, diameter 47 mm) attached to Atmoservice PNS-15 aspirators (produced in Poland under the licence of low-volume sampler LVS 3.1, Comde-Derenda GmbH, Germany) with flow rates of 2.3 m^3^/h. Since indoor samples of PM_2.5_ were derived from the sum of PM1 and PM_2.5_-1 concentrations that were collected at two stages of the impactor, larger uncertainties may be associated with the indoor composition of this fraction. According to the manufacturer’s information, the uncertainties of a standard sampler are below 1%, while the impactor is characterized by uncertainties below 2.8%.

Before and after sampling, the membranes and filters were conditioned (temperature 20 ± 1 °C, relative humidity 50% ± 5%) for 48 h and weighted with a microbalance precision of 1 µg (MXA5/1, RADWAG, Poland).

The sampling position in the classrooms was set at the height of the breathing zone of the children (*i.e.*, about 0.8–1.0 m above the floor).

### 2.3. Analytical Method

Concentrations of 11 trace elements (As, Cd, Cr, Cu, Fe, Mn, Ni, Pb, Sb, Se and Zn) in PM_2.5_ were determined. The analysis of these elements was done using atomic absorption spectrometers with an acetylene-air flame Avanta PM and a graphite furnace Avanta GM (GBC Scientific Equipment Pty Ltd., Melbourne, Australia). The standard solutions of the trace elements were obtained from Merck, Germany. Solutions of different concentrations were prepared by standard dilution 1000 mg/L (CeriPUR®). The collected PM_2.5_ samples were mineralized in a mixture of concentrated (ultra-high purity) HNO_3_ (8 cm^3^) and H_2_O_2_ (2 cm^3^) according to PN-EN 14902 standard [21]. Ultrapure acid and hydrogen peroxide for trace analysis from Sigma Aldrich TraceSELECTultra® were used, which allowed a clear solution to be obtained. Samples of the filters with dust were digested in the microwave system (Multiwave PRO, Anton Paar) at a temperature of 260 °C, and the pressure was 60 bar. The mineralization product was put into a 25 cm^3^ measuring flask and unionized water was added to complete the flask volume. Such solutions were filtered by the DigiFILTER system, PerkinElmer (0.45 µm). To check the accuracy and precision of the extraction protocol, SRM NIST 1649a Urban Dust and NIST 1648 Urban Particulate were used. The limits of detection for the method, found by analysing blanks (clean filter substrates) according to PN- EN14902, were 0.3 ng/m^3^ for As, 0.25 ng/m^3^ for Cd, 3.1 ng/m^3^ for Cr, 1.4 ng/m^3^ for Cu, 5.8 ng/m^3^ for Fe, 1.5 ng/m^3^ for Mn, 0.25 ng/m^3^ for Ni, 0.5 ng/m^3^ for Pb, 0.45 ng/m^3^ for Sb, 1.0 ng/m^3^ for Se and 5.0 ng/m^3^ for Zn.

### 2.4. Factor Analysis and Source Identification

The contamination levels of As, Cd, Cr, Cu, Fe, Mn, Ni, Pb, Sb, Se and Zn in the studied PM_2.5_ samples were evaluated using EF analysis. The enrichment factor *EFx* for the element *x* is defined as:

$$\;\;\;\;\;\;\;\;EF_{x}=\frac{{\left(C_{x}/C_{ref}\right)}_{PM2.5}}{{\left(C_{x}/C_{ref}\right)}_{crust}}$$

where *C_x_* and *C_ref_* are the concentrations of the element *x* and the reference element, and ${\left(C_{x}/C_{ref}\right)}_{PM2.5}$ and ${\left(C_{x}/C_{ref}\right)}_{crust}$ are the proportions of these concentrations in the PM and in the Earth’s crust, respectively. In this study, Fe has been used as an indicator for the main source of the Earth’s crust composition [15,22,23,24,25,26]. Fe is not the only possible choice, but it is the one widely used. Other results together with Fe encounter Mn and Ti [24]; Al, Si, Ca and Ti [25]; Al, Ti, K and Mn [23] which can be used as a reference elements. The chemical composition of the upper continental crust was taken from Wedepohl [27]. The EF can be calculated to evaluate the degree of enrichment of a given element compared to the relative abundance of that element in a crustal material. Values lower than 10 indicate that the element investigated has a significant crustal source (soil), while EF values higher than 10 are ascribed to elements with mainly a significant anthropogenic origin. An EF value between 10 < EF < 100 can be considered moderately enriched, while the value >100 can be deemed highly enriched [15].

In order to obtain a reliable identification of different sources contributing to fine particles, PCA was used. This technique involves a mathematical procedure that transforms a number of possibly correlated variables into a smaller number of non-correlated variables called “principal components”. It is commonplace for the sum of the variances of the first few principal components to exceed 70% of the total variance of the original data. Therefore, in many cases, the first two or three components are enough to develop a deeper understanding of the driving forces that generated the original data and/or to demonstrate the correlation between the original variables [28,29].

### 2.5. Statistical Analyses

All statistical analyses, including univariate and multivariate analysis (including Pearson correlation regression, the *t* test, and the Kolmogorov-Smirnov and Lilliefors tests) as well as principal component analysis, were performed using the statistical package Statistica 10 (StatSoft). Non-parametric tests were undertaken to confirm the parametric results—that is, the corresponding non-parametric tests led to the same conclusions of significance/non-significance as the parametric tests. Throughout the study, a *p* value of <0.05 was considered to indicate statistical significance.

## 3. Results and Discussion

The indoor and outdoor concentrations of PM_2.5_ were measured at all the nursery schools during winter and spring. The Upper Silesia region, compared with other EU countries as well as other Polish regions, is characterized by relatively high levels of PM. Although the last three decades of economic changes forced the greatest Polish drop in industrial air pollution, the old steel works, cookeries, and coal mines, together with re-suspension processes from urban surfaces and road traffic, are responsible for high concentrations of ambient dust [30,31]. Other investigations were done in a winter heating season, confirming that the emissions from combustion of fossil fuels for energy production (especially municipal) causes very high PM concentrations [32]. However, pollutants emitted from industrial coal combustion processes have been significantly reduced, while the emissions from small-scale combustion utilities, such as domestic boilers, have become particularly dangerous. The hazard of domestic sources originates from the low quality of fuels (coal, biomass, culm or even refuse) used for heating, especially during winter. According to the dominant role of coal combustion in the region, diverse trace-element content between winter and spring are expected. At the same time, the nursery school buildings differ in location, which can influence the trace-element levels, especially between urban and rural sites.

### 3.1. Mass Concentrations

**Figure 2:**
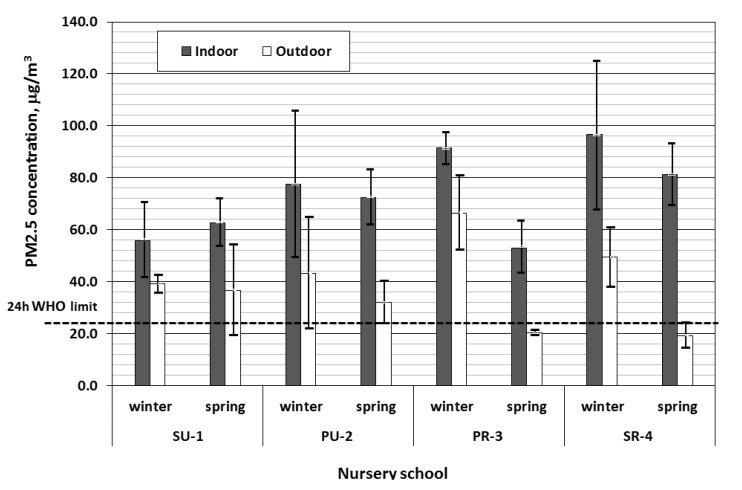
PM_2.5_ concentration measured indoors and outdoors.

The indoor and outdoor PM_2.5_ mass concentrations for selected nursery schools measured during winter and spring (Figure 2) varied from 19.37 to 66.36 µg/m^3^ in outdoor samples and from 53.09 to 96.67 µg/m^3^ in indoor samples. The average concentrations of indoor and outdoor samples were 73.90 µg/m^3^ and 38.36 µg/m^3^, respectively. The outdoor average concentrations of PM_2.5_ are typical for the Upper Silesia region [31,33]. The indoor average concentrations of PM_2.5_ samples collected in Portuguese preschools were found to be at similar levels during the occupation of children at rural nursery schools: 100 ± 71 µg/m^3^ [34]. Meanwhile, the PM_2.5_ hourly average concentrations varied from 9.03 to 28.06 µg/m^3^ [10] and from 19.70 to 34.69 µg/m^3^ [3,10], respectively, in the classrooms located in rural and urban nursery schools. Meanwhile, in Swedish preschools equipped with mechanical ventilation, the PM_2.5_ concentrations were significantly lower, between 3.2 and 9.3 µg/m^3^ [11].

The highest indoor concentrations were found during winter in the rural nursery school (SR-4) situated next to the highway, while the lowest were observed during spring in the rural nursery school (PR-3). The maximum and minimum outdoor levels of PM_2.5_ were also found at rural sites; however, the lowest mass concentrations in spring were found outside the rural nursery school (SR-4) situated next to the highway, while the highest were observed at the rural site (PR-3) in winter. The trend with maximum and minimum PM_2.5_ levels at rural sites is specifically connected with the fact that heating in Poland is still based on coal combustion, which produces an increase of particulate emission from heating in winter and a decrease in spring. Domestic sources are particularly dangerous during winter because they use low quality or other fuels to heat the coal: biomass, culm or even refuse. At urban sites, the domestic sources are less important while automobile emission is a major source of PM_2.5_.

The discrepancies between indoor and outdoor (I/O) PM_2.5_ concentrations were significantly different during spring (*p* = 0.02 and *p* = 0.006 respectively at urban and rural sites). During winter, I/O concentrations were different at rural sites (*p* = 0.02), but at urban sites the difference was not significant (*p* > 0.05).

Analysing the PM_2.5_ mass concentrations according to location (urban or rural site), during winter the PM_2.5_ concentrations were higher at rural sites, while during spring the urban sites were more polluted. However, the discrepancies between PM_2.5_ (indoor and outdoor) concentrations at urban and rural sites are not significant (*p* = 0.08–0.62). Other worldwide research generally points out higher concentrations of PM at urban sites compared to rural sites [3,4,10]. However, in Poland, during winter, domestic sources use low-quality coal, biomass or even refuse for heating. Thus, rural areas are often characterized by lower air quality than urban areas.

### 3.2. Trace Element Concentrations

The concentrations of As, Cd, Cr, Cu, Fe, Mn, Ni, Pb, Sb, Se and Zn in the PM_2.5_ samples collected from urban and rural nursery schools during winter and spring are presented in Table 1 (ng/m^3^) and Table A1 (µg/g). The total concentrations of trace elements distributed in PM_2.5_ were different according to season and location. According to the season, higher indoor total concentrations at urban and rural sites were determined during spring. The higher concentrations of elements during the warm period presumably suggest a stronger influence from soil and road dust re-suspension in the warm months [35,36], but this trend was not significant—*p* = 0.31 and 0.54, respectively, in urban and rural sites. The relation between outdoor concentrations was different at urban and rural sites. In urban areas, the total concentration of trace elements was higher during spring (*p* = 0.57) while at rural sites winter was characterized by a higher concentration of the sum of eleven trace elements (*p* = 0.62).This is in agreement with the hazardous role of domestic sources, which see the use of low-quality fuels for heating (coal, biomass, culm or even refuse), especially during winter.

Wang *et al.* [37] proposed grouping trace elements into two major groups: earth crust elements or soil tracers and anthropogenic tracers. Earth crust elements comprised Na, Al, K, Mg, Ca, Fe, Ti and Mn, while V, Cr, Cd, Ni, Cu, Pb, Zn, As, Sn and Se could be considered partially natural and partially anthropogenic in origin, depending on the source region and travel path of the air mass. Following this proposal, Fe and Mn could be included into the Earth crust elements while other elements could be considered partially natural in origin and partially anthropogenic.

In the present study, Fe, Cr and Zn were recorded as having the highest concentration among the trace elements collected at all sites. They accounted for over 85% of 11 target trace elements, while As, Cd, Cu, Sb and Se had lower concentrations and accounted for 5% of the determined trace elements. At urban sites as well as at rural sites, during winter the highest concentration indoors and outdoors presented Fe, while during spring (at rural sites) the highest concentration revealed Cr. There were no significant differences (*p* > 0.05) between Fe concentrations at urban and rural sites during both seasons. Meanwhile, during spring, Cr presented significantly (*p* = 0.0008–0.03) higher concentrations at rural compared to urban sites in indoor as well as in outdoor air. At the same time, during both seasons, the levels of Fe at rural sites in the classrooms were significantly (*p* = 0.01–0.03) higher than outside. Soil particles can be tracked in on shoes or clothing from the outdoors, contributing to indoor–outdoor ratios (I/O) in the range 0.92–3.3 (average = 1.7). Higher I/O ratios from 1.2 to 3.3 were obtained at the first floors of nursery schools, as Fe is the major element in the Earth’s crust and hence has the ability to influence the level of Fe in the indoor dust [15]. Besides being transported by children (on shoes and clothes), particles from outside with high Fe loadings could have contributed to the indoor PM_2.5_ concentrations through the re-suspension of particles [10]. As a major source of Cr in PM_2.5_ during summer, the authors [38] in the literature review enumerated motor vehicles, coal-fired boilers and natural soils. Other encounter sewage sludge incineration [29,37]. and solid waste [39]. Petaloti *et al.* [35] suggested that the significantly higher concentrations of crustal and traffic-related elements (Ti, Mn, P, Cr, K, Al, Fe, Sr and Cu) in the warm period are probably influenced by soil and road dust re-suspension in the warm months. Because the high level of Cr during spring was not significantly different between the rural sites (PR-3 in the village, and SR-4 next to the highway), the source coming from soil re-suspension seems to be the most influential. In selected location, it most likely represents emissions from sewage sludge dumping on agricultural fields, which took place at this time at the rural sites [28,35].

**Table 1:** Winter and spring PM_2.5_ trace elements concentrations, ng/m^3^.

Trace Elements	Winter								Spring							
	Urban ( *N* = 19)				Rural ( *N* = 25)				Urban ( *N* = 19)				Rural ( *N* = 21)			
	Indoor	SD	Outdoor	SD	Indoor	SD	Outdoor	SD	Indoor	SD	Outdoor	SD	Indoor	SD	Outdoor	SD
As	6.60	4.61	3.05	5.74	4.93	2.89	1.97	2.04	0.16 *	0.11	0.08 *	0.08	1.52	0.98	0.35 *	0.44
Cd	13.26	2.96	6.48	1.88	15.95	2.08	8.04	1.01	17.92	2.66	9.02	2.49	18.57	1.36	9.91	2.90
Cr	50.58	47.61	7.79	3.17	70.16	31.04	25.65	17.01	244.62	79.12	128.20	68.27	448.32	23.22	244.28	65.03
Cu	25.23	12.62	11.43	15.13	20.06	4.71	7.95	4.44	24.23	27.82	5.14	3.40	6.61	3.36	1.40	1.50
Fe	442.75	154.36	258.47	281.61	508.94	164.61	250.23	68.53	437.15	229.55	295.06	242.40	342.98	142.90	133.07	101.00
Mn	23.17	8.73	10.04	5.09	33.28	14.82	14.14	2.14	23.68	4.98	12.37	4.61	21.26	7.24	11.07	2.20
Ni	22.73	17.37	8.24	4.63	15.43	10.42	2.10	1.73	27.91	28.45	3.55	2.10	11.22	7.78	7.31	8.37
Pb	24.15	20.85	29.82	21.76	48.41	5.55	50.32	11.97	36.35	25.08	26.65	12.30	16.30	8.36	14.59	13.43
Sb	1.44	1.82	0.25 *	0.07	3.45	1.45	0.41 *	0.32	1.21	0.37	0.35 *	0.17	0.39 *	0.22	0.26 *	0.15
Se	1.45	2.01	0.51 *	0.45	0.38 *	0.29	0.73 *	0.69	1.12	0.56	0.42 *	0.31	0.62 *	0.44	0.64 *	0.47
Zn	226.56	62.14	97.42	61.51	243.40	24.70	155.15	28.54	212.13	45.14	90.19	20.84	174.82	18.72	56.16	22.07
Total	837.91	335.08	433.49	401.04	964.40	26_2.5_5	516.69	138.42	1 026.48	443.82	571.02	356.98	1 042.62	214.57	479.04	217.55

*N*: number of simultaneous indoor and outdoor daily sessions. *: below limit of detection (<LOD).

In Table A2 the Pearson’s correlation matrix between the trace elements in PM_2.5_ samples collected inside and outside sampling sites are summarised. The possible sources around sampling sites can be qualitatively identified from the single correlation coefficients. Specifically, there is a clear correlation between Pb-Zn (*r* = 0.93), As-Cu (*r* = 0.90), Fe-Mn (*r* = 0.82), Cd-Cd (*r* = 0.74), as well as between Cu, Fe, Mn, Pb and Zn. This suggests a possible common origin for this species.

### 3.3. Enrichment Factor Analysis

**Figure 3:**
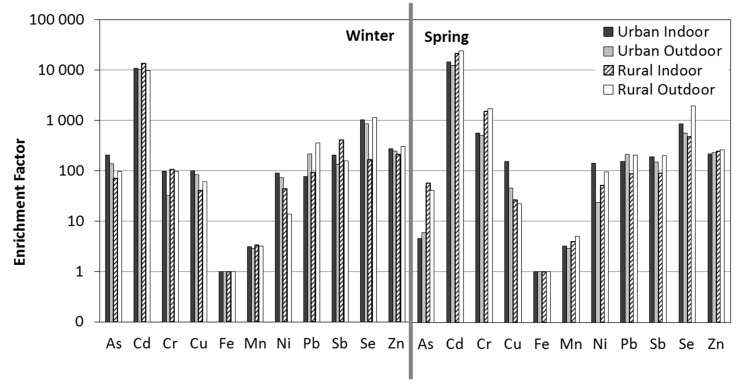
EF analysis using Fe as a reference element.

EFs were calculated to present the degree to which the selected trace elements in PM_2.5_ are enriched relative to the crustal and non-crustal sources. Within this work, EFs were calculated in relation to Fe, as a consequence EF_Fe_ = 1. These 11 trace elements could be divided into three groups according to the calculated results: a highly enriched group with EF > 100, an intermediately enriched group with EF between 10 and 100, and a slightly enriched group with EF < 10. As Figure 3 shows, Cd exhibited the highest EFs (≥10,000). High EFs (>100) during both seasons showed Sb, Se and Zn as well as Pb in outdoor air. During winter, As presented a high EF, while Cr showed a high EF during spring. The high enrichment of these elements suggests that the dominant sources for these elements were non-crustal, and a variety of pollution emissions contributed to their loading in PM_2.5_. The moderate EFs during both seasons generally presented Cu, Ni and As at rural sites, while Cr showed moderate EF during winter. During spring, Mn and As presented low EFs as well.

Taking the atmospheric contribution of As into consideration, it is linked to fossil fuel combustion (traffic, domestic heating, natural gas, *etc.*) [40]. The concentration of this contaminant was lower during spring (*p* = 0.03–0.3) especially at rural sites, suggesting the anthropogenic contributions are determined mainly by domestic heating.

Cd, like As, originates from coal combustion [35], and other sources are steel, plastic and pigment production as well as tire wearing [24]. At both sites, little marked variation between seasons occurred. The tendency for the Cd contribution in the atmosphere to be slightly higher during spring indicates the possible role of soil re-suspension processes.

Cr reflects a variety of pollution sources, in particular coal burning and sewage sludge incineration [37]. The elevated EFs during spring in comparison to winter point to the emissions from sewage sludge dumping on agricultural fields which took place at this time at the rural sites.

Cu is emitted from smelting furnace burning as well as vehicle emissions (diesel combustion and brake lining wear) [24].

Pb and Zn are traditional tracers of vehicle emissions [37]. Although Pb is no longer added to petrol, it is found as a trace element in various fuels. It is also produced in domestic boilers because in rural aerosols, the high enrichment would require hot sources. Finally, it should be discharged from soil, where it accumulates over decades and where it will partly remain. In addition, Zn is found to be one of the major components of road dust as a result of the accumulation of Zn emitted from tires, motor oil and the use of motor vehicle brakes [15].

Low EFs of Mn point to crustal matter [22,23]; however, the manganese tricarbonyl compound is used as an additive in unleaded petrol to enhance automobile performance [29]. Moreover, in outdoor air Mn is moderately correlated with Pb (*r* = 0.53) and Zn (*r* = 0.67), which are attributed to vehicular emission.

Ni is present as a trace element in petroleum, so if the anthropogenic origin is considered, its presence in the atmosphere is primarily related to fossil fuel combustion (carbon and fuel oil) in the production of electricity and heat, as well as traffic emissions [40]. It is also emitted from the burning of lubricant oil [29].

Sb and Se with large EFs had rather low concentrations, thus suggesting the significant contribution of anthropogenic sources for Sb and Se. The shared source of Sb and Se is coal-fired boilers [38,41]. Separately, the main source of Se is oil-fired power plants [38] while Sb is emitted from long-term mining and smelting. Additionally, antimony sulphide is widely used to enhance frictional stability properties and to reduce vibrations in vehicle brake pads [42]. Thus, airborne Sb emissions are an important concern. Fujiwara *et al.* [43] showed the presence of elevated concentrations of antimony in road dust, as well as traffic-related elements like Pb and Cu, correlated well with heavy traffic areas in Buenos Aires, Argentina.

Combined with enrichment factor analysis (Figure 3) and Pearson’s correlation coefficients (Table A2), the markers of various sources can be identified as:

(1) As, Cu, Pb and Zn for coal combustion and vehicle emission;
(2) Fe and Mn for crustal origin (according to low EF);
(3) Cd and Cr for soils contaminated by sewage sludge dumping (according to higher EFs during spring);
(4) Ni, Sb and Se for fossil fuel combustion (carbon and fuel oil).

### 3.4. Principal Component Analysis

In a further attempt to determine the associations between elements generated by diverse emission sources, PCA was applied to the outdoor dataset of trace element concentrations (ng/m^3^) to assess the sources responsible for the observed pollution levels. Factor loadings, with a Varimax rotation for PM_2.5_, are calculated and presented in Table 2. Although there are no well-defined rules for the number of factors to be retained, usually either factors that are meaningful or factors with eigenvalues larger than unity are retained. In this work, three factors with eigenvalues larger than unity were extracted (Table 2), and they accounted for 75% of the total explained variance of outdoor dataset. For the purposes of the following discussion, loadings with absolute values larger than 0.5, in the range of 0.2–0.5 and lower than 0.2 are characterized as high, moderate and weak, respectively (weak loadings are suppressed in Table 2).

**Table 2:** Principal component loadings and variance explanation for PM_2.5_.

Trace Elements	PC1	PC2	PC3
As	**0.888**		
Cd		**0.889**	0.301
Cr	−0.311	**0.872**	
Cu	**0.920**	−0.222	
Fe	**0.831**		
Mn	**0.734**		0.317
Ni		**0.518**	−0.363
Pb	**0.694**		0.455
Sb			**0.880**
Se	0.230		**0.742**
Zn	**0.757**	−0.241	0.476
% variance	37.2	18.6	19.5

Component loadings lower than 0.20 are suppressed. Loads larger than 0.5 (in absolute values) are in bold.

The first component (PC1) contributed 37.2% of the total variance. The high loading of As, Cu, Fe, Mn, Pb and Zn in one factor provides an indication of possibly mixed sources, *viz*., anthropogenic and geogenic sources. It is important to point out that the matrix of PC1 loads confirms some conclusions inferred by the previous analysis of the correlation coefficients. However, separating this complex pollution into individual components is difficult. Since Zn is correlated with Pb (*r* = 0.93) and with Cu (*r* = 0.72), the presence of Zn-Pb-Cu in one factor provides an indication of possible traffic sources [24,29,40]. The high correlation between As and Cu (*r* = 0.90) could indicate coal combustion, especially for domestic heating purposes [38,41]. Meanwhile, Mn and Fe (*r* = 0.82) might originate from crustal matter [22,23].

The second component (PC2) was accounted with high loading of Cd, Cr and Ni with 18.6% variance. The presence of a high load of Cd and Cr found with correlation coefficient (*r* = 0.74) and high EFs of all three elements could indicate a contribution to re-suspension of soil dust contaminated by sewage sludge dumping on agricultural fields [29,37] which took place at this time at the rural sites.

The third component (PC3) contributed about 19.5% which includes Sb and Se. The moderate correlation elements of elements (*r* = 0.55) together with large EFs that had rather low concentrations indicates anthropogenic sources. Simultaneously Cd, Mn, Pb and Zn present moderate loadings in this factor, meaning PC3 most probably originated from a variety of vehicular emissions [40,43].

## 4. Conclusions

PM_2.5_ samples were collected outside and inside four naturally ventilated nursery schools located in the area of Gliwice, Poland. The samples were analysed for As, Cd, Cr, Cu, Fe, Mn, Ni, Pb, Sb, Se and Zn concentrations to examine the variation in location (indoor, outdoor), urbanization (urban, rural) and season (winter, spring). The major findings of this study are:

- Indoor PM_2.5_ concentrations exceeded the WHO guidelines regardless of the season and location. Outdoor PM_2.5_ concentrations were significantly lower than those indoors; however, they met the WHO guidelines only in spring at rural sites.
- An anthropogenic origin revealed most trace elements; however, the most enriched (EF > 100) elements in indoor PM_2.5_ were Cd, Se, Sb, Zn and—additionally—Cr in spring.
- The PCA results show that anthropogenic emissions are the most important sources of trace elements in outdoor PM_2.5_ aerosols. The highest relative contribution to the sample variance was 37.2% and included As, Cu, Fe, Mn, Pb and Zn.

The study showed that preschool children can be exposed to trace elements from a variety of anthropological sources. Extensive investigation of the presence of trace elements in and around the nursery school environment is crucial to reduce children’s exposure to such elements. Other studies should be conducted in order to evaluate whether or not there is a causal relationship between trace element exposure and health symptoms in nursery schools, and whether this may adversely affect children’s attendance.

## Appendix

**Table A1:** Winter and spring PM2.5 trace elements concentrations, µg/g.

Trace Elements	Winter								Spring							
	Urban( *N* = 19)				Rural( *N* = 25)				Urban( *N* = 19)				Rural( *N* = 21)			
	Indoor	SD	Outdoor	SD	Indoor	SD	Outdoor	SD	Indoor	SD	Outdoor	SD	Indoor	SD	Outdoor	SD
As	191.63	99.15	48.61	27.72	96.12	41.10	30.97	12.76	4.70	2.71	2.94	1.25	52.80	28.38	16.45	11.64
Cd	518.78	181.71	187.02	64.01	924.31	268.56	159.31	56.33	754.69	147.10	306.58	109.21	1027.82	291.24	493.12	136.50
Cr	1612.29	444.54	197.74	78.14	2508.63	1063.52	539.49	272.60	10,117.01	3588.64	4352.67	1298.21	25,080.53	10,706.97	12,096.61	2916.78
Cu	672.39	176.23	210.49	113.59	395.51	124.85	136.88	44.65	1123.43	814.24	159.13	53.92	175.69	86.86	64.73	21.53
Fe	14,433.14	2149.43	5520.24	1572.40	21,177.97	12,938.67	4879.95	1831.58	15,863.28	6212.33	7551.46	2217.47	14,629.79	1752.02	6199.13	1980.55
Mn	773.80	243.02	266.91	154.34	1204.20	551.22	269.26	59.32	874.08	232.22	374.62	81.60	981.26	276.63	542.78	45.19
Ni	786.04	283.07	244.89	157.16	549.41	198.77	40.56	28.01	1349.81	703.23	106.78	63.87	451.67	280.11	355.26	188.04
Pb	605.94	292.59	659.46	206.78	1063.69	379.66	967.59	254.10	1339.55	478.26	881.83	496.96	691.58	336.31	702.62	299.85
Sb	29.88	14.21	7.32	3.16	86.59	40.09	7.68	5.51	30.42	10.10	11.26	5.06	12.90	4.67	12.54	6.31
Se	39.63	22.56	12.82	6.64	9.22	5.77	14.87	7.73	36.96	14.42	11.31	3.68	18.47	6.61	32.77	14.39
Zn	6646.99	3451.87	2274.36	598.42	7447.87	1278.67	2497.93	289.89	5769.98	752.12	2875.07	874.32	6002.56	1807.12	2745.78	915.78
Total	26,310.48	8788.39	9629.86	5208.36	35,463.52	25,090.88	9544.49	3188.48	37,263.91	17,070.39	16,633.63	9437.53	49,125.07	16,986.94	23,261.78	9116.56

*N*: number of simultaneous indoor and outdoor daily sessions.

**Table A2:** Correlation coefficients between PM_2.5_ and different trace elements collected inside (in) and outside (out) urban and rural sites.

Location	in												
in		PM2.5	As	Cd	Cr	Cu	Fe	Mn	Ni	Pb	Sb	Se	Zn
	PM2.5	1.00	0.12	0.09	−0.30	0.19	***0.75***	*0.62*	−0.07	0.39	*0.50*	0.28	*0.47*
	As		1.00	− *0.63*	− *0.54*	0.18	0.26	0.21	0.11	0.15	0.46	0.22	*0.62*
	Cd			1.00	*0.61*	−0.45	0.03	0.27	−0.33	−0.30	−0.10	−0.02	−0.28
	Cr				1.00	− *0.53*	−0.27	−0.25	−0.19	− *0.54*	− *0.60*	−0.14	− *0.51*
	Cu					1.00	0.16	0.12	***0.77***	*0.67*	0.23	0.16	0.05
	Fe						1.00	***0.75***	0.10	0.15	0.32	0.20	*0.50*
	Mn							1.00	0.16	0.11	0.46	−0.14	0.30
	Ni								1.00	0.30	−0.15	−0.19	−0.09
	Pb									1.00	*0.57*	0.17	0.28
	Sb										1.00	0.26	*0.50*
	Se											1.00	0.37
	Zn												1.00
	**out**												
out	PM2.5	1.00	*0.61*	−0.27	− *0.59*	*0.62*	*0.55*	*0.57*	−0.18	***0.76***	−0.05	0.01	***0.80***
	As		1.00	−0.06	−0.33	***0.90***	*0.57*	0.46	−0.15	*0.57*	−0.04	0.21	*0.61*
	Cd			1.00	***0.74***	−0.26	−0.17	0.03	0.24	−0.05	0.31	0.32	−0.07
	Cr				1.00	− *0.47*	−0.31	−0.17	0.32	− *0.49*	−0.02	0.02	− *0.52*
	Cu					1.00	***0.76***	*0.57*	−0.23	*0.65*	0.10	0.26	***0.72***
	Fe						1.00	***0.82***	−0.25	*0.48*	0.15	0.22	*0.60*
	Mn							1.00	−0.36	*0.53*	0.29	0.28	*0.67*
	Ni								1.00	−0.14	−0.13	−0.17	−0.26
	Pb									1.00	0.38	0.39	***0.93***
	Sb										1.00	*0.55*	0.41
	Se											1.00	0.39
	Zn												1.00
**Location**	**out**												
in		PM2.5	As	Cd	Cr	Cu	Fe	Mn	Ni	Pb	Sb	Se	Zn
	PM2.5	*0.54*	0.38	−0.23	−0.42	0.39	0.45	0.40	− *0.58*	0.46	−0.12	0.13	*0.49*
	As		0.34	− *0.53*	− *0.55*	0.42	0.12	−0.07	−0.01	0.30	0.04	0.05	0.35
	Cd			*0.63*	*0.62*	−0.35	0.01	0.23	−0.22	−0.35	−0.07	0.02	−0.30
	Cr				***0.93***	− *0.53*	−0.32	−0.21	0.15	− *0.64*	−0.17	−0.09	− *0.65*
	Cu					0.42	0.19	−0.02	−0.11	0.35	−0.14	−0.15	0.24
	Fe						*0.48*	0.30	−0.31	0.23	−0.08	−0.13	0.22
	Mn							0.20	−0.37	0.04	−0.10	−0.24	0.12
	Ni								0.02	−0.09	−0.29	− *0.49*	−0.21
	Pb									***0.82***	0.17	0.15	***0.77***
	Sb										*0.48*	0.37	***0.80***
	Se											0.26	0.32
	Zn												*0.51*

Bold entries indicate “strong” correlations (larger than 0.7 in absolute value) and italic entries indicate significant correlation at the *p* level 0.05.
